# Supplementary material for: I did not scream. i could not; i was terrified. i just followed them. . .i blocked my mind. then they all raped me: A narrative inquiry on the onset of tonic immobility among women rape victims in Nigeria
Source: PLoS One. 2024 Feb 5;19(2):e0278810. doi: 10.1371/journal.pone.0278810 (PMC10843104; doi:10.1371/journal.pone.0278810)
Supplement: S2 File — (DOCX) [file pone.0278810.s002.docx]

**EXTRACTS FROM TRANSCRIPTS OF SURVIVORS’ STORIES**

**Participant (P1, 26-30).**

It happened on a Thursday. I can’t really recall that day, but I was on my way coming back from the market, I went to deliver something rather, but the people closed so I had to cross back. So, I saw those boys. I first saw one of them, so I thought maybe he was urinating or something, I moved on to cross a railway path. So immediately I crossed over, and the guy came from behind and tapped me. I was like, please I do not know you. He replied that he me well from my street. He called my brother’s name; he mentioned one or two things about me that shows that he knows me. So, I like was still denying him that no, I don’t know you. Then later he called this other gang, four guys, so they came out from where they were, just surrounding. *It was still early in the evening that day after work, on my way to the bus stop, when four men approached me from nowhere. One of them showed me a big/shining knife that he had with him, while another one of them asked me to follow them. Immediately when I saw that knife, I was shocked and froze there and then. My whole body just went weak. I did not know what to do. I did not scream; I could not scream; I was too terrified. “I just complied with what they asked me to do. I just followed them; I do not know how...I blocked my mind. I think they all raped me...”* That was how they attacked me. They took me under a bridge. It was there under the bridge that they raped me.

**EXTRACTS FROM TRANSCRIPTS OF SURVIVORS’ STORIES OF THEIR EXPERIENCE FROM TONIC IMMOBILITY**

**PARTICIPANT 2 (26-30).**

*I am a hotel receptionist. On this night, a guest called that he had a problem with his room, so I went to assist him. When I entered his room for him to show me what was wrong so I could help him, he grabbed me from behind, held me tightly, and pushed me further into the room. He threatened me with a gun and a knife and said if I did not cooperate with him, he would use both on me. My body went cold. I was confused, afraid, and just felt very weak and could not move; my knees could not carry me. He pushed me onto the bed. That was when he raped me. I just looked at him and could not even scream or fight him off.* He had even collected my phone and switched it off. I was even calling on Jesus. He ordered me to shut my mouth up. He said that the manager knew him and so even if he was told of the rape, would do nothing. When I saw the knife, I started crying, shouting Jesus, Jesus, help me, don’t let me die, don’t let me die. I wanted to pull back from him, but he said, I will shoot you, I will shoot you either in the chest or my forehead here (pointing to her forehead). I begged him in the name of God not to do it. He asked me if I wanted to have sex with him, I said no. He brought out a knife and gun again and threatened me with them. I was very afraid. My body felt different. He forced me to sex. He forced me, I had said no. I even vomited. I was beside myself. It did not feel that I was undergoing such a thing. He asked me to lick his prick. But I was not there, my mind had gone to heaven, only my body was on earth.

**EXTRACTS FROM TRANSCRIPTS OF SURVIVORS’ STORIES OF THEIR EXPERIENCE FROM TONIC IMMOBILITY**

**PARTICIPANT 3 (26-30).**

**Theme 1. Onset of tonic immobility prior to rape due to perceived imminent danger.**

Ok, the truth is that I hate talking about it but since you had asked and I agreed, I will try my best to talk about it again. Know that I work by the day and sell cooked food in the evenings. So, *as a food vendor, I sell food around. Since my husband lost his job, I have been selling cooked food. So, this man I have known for a while called me to buy food from me. He asked me to get a plate from his kitchen, and that is where he attacked me. He brought out a kitchen knife from a kitchen drawer and pointed it at me. It was unbelievable, I wish I had done something to stop him, but I could not. I was rooted there. I could not move or say anything. I obeyed all his commands. He raped me. I was weak, shocked, and ashamed, but I did not feel anything about what he did to me.* That is how it happened. First, it was so sudden, and I never expected this. In fact, I never suspected that that was going to happen. So, it came suddenly, and I just didn’t know what to do. I was stunned; I was paralyzed with fear on seeing the gun and I could not even open my mouth. I could not utter a word; I simply followed his instructions without even knowing what I was doing”.

**Theme 3: self-loathing as meaning attached to TI experience.**

When I think of its reactions in terms of defense or all that, I had none. I just complied, I just felt, I was out of myself. I just felt I became like a log of wood, and I didn’t feel anything. I just felt paralyzed all over my body. I kind of remembered, while he was doing it, I felt disconnected even, so that I would not be in touch with whatever he was doing. I just hated the ‘whole thing’. I didn’t even want to accept that he was doing this to me. Sincerely, it was a traumatic experience, and something I wished I never had to experience but unfortunately it happened. *After it (rape), I just felt as if the whole world knew that I gave in to that without a fight or a cry for help. I felt that my self-worth was reduced, as if everyone knew what had happened to me.  And that kind of made me ashamed of myself, lose my confidence, and feel unworthy. I had let myself down, obviously.*

*Feeling about myself, I will say that after what happened, I feel dirty, ashamed, unworthy, and that something valuable had been taken away out of me. So, I did not know whether I was just ashamed to tell you the truth, but I felt dirty, and I hated everything about myself after that.*

**EXTRACTS FROM TRANSCRIPTS OF SURVIVORS’ STORIES OF THEIR EXPERIENCE FROM TONIC IMMOBILITY**

**PARTICIPANT 4 (26-30).**

I was walking along the street at night, but unfortunately, the mistake I made was that I passed the route people don’t pass at night. In fact, I had been warned earlier not to take that route but since it’s the fastest way to home, I did. While walking home, I noticed that two men were coming behind me. At first, I wasn’t scared, as had I thought they were also taking the route. No other person was on that same route with me, except them. I noticed that whenever I stopped, their movement also stopped and they increased their speed, as I increased mine and eventually caught up with me before I could get a lit area. One of them grabbed me and a had his other hand over my mouth. I screamed and screamed, the other one held me down. I was being beaten, I tried fighting back but I couldn’t. I was so afraid; I was so confused and wondered what these people wanted from me. I never realized that they wanted to rape me. I became stressed, so scared, I couldn’t even scream, there was nothing happening around, people were not passing by since the place was dark……. It was very late at night. The next thing, one of them suggested that they take turns in raping me and told the other guy to hold my hands down. I was suddenly overwhelmed with tiredness that I couldn’t even fight so I had to let them do whatever they wanted to do. At that point*, they pushed me down. It seemed like a long while before they started ripping off my clothes, tearing my bra, and doing all sorts of things to my body including running a razor blade over my naked body that I realised this was the end for me. So, I was terribly scared by then, but unable to shout. I could not even move my body. I could not even struggle; I just lay there on the ground and closed my eyes. Each one of them raped me after the other and I was just lying there like a rod.* It wasn’t easy; it was a bad experience for me. The first one finished [with the rape] and was like laughing while I was in tears. Nobody was there to help me. So, I just resigned and left them to continue.

**Theme 5: Divine intervention as the meaning made of tonic immobility.**

*Even though lying there paralysed and not able to do anything was bad and terrible, it proved to me that God loves me. I believe that it was God that was working for me.* *He confused my assailants to think I was dead so that they could stop the rape. I thank God. The men who assaulted me threatened to deal with me, but God confused them. They left me after raping me, they could have killed me; I give God the glory. He is truly a living God.* **(P4, 26-30).**

**EXTRACTS FROM TRANSCRIPTS OF SURVIVORS’ STORIES OF THEIR EXPERIENCE FROM TONIC IMMOBILITY**

**PARTICIPANT 5 (26-30).**

It was supposed to be a friendly outing after which *my friend’s boyfriend was to take me home, but then he first asked me for sex, and I refused. He got violent and started hitting me and threatening to throw me out of his room. This was around 2 am. I knew that I was stuck there. He started beating me. I first tried to fight him, but he did not stop beating me. Suddenly I could not move, my arms could not fight him. I could not scream either. I could no longer move but knew what he was doing to me. He pulled down my clothes, ripped my underwear, and did it*. Going back, what happened was that I wasn’t aware of whatever was going to happen at the venue. But before then, I made it clear that I wasn’t for any sexual game, and I was assured that it was going to be a friendly meeting. When we got there and there, there were three guys and three of us ladies. So, I sort of became scared. But since I was already assured of what to expect, I tried to relax as we got into the hotel. We got into this hotel room, and after a while, they said to me, oh you are following this guy. I said but this is not what we agreed. One of the girls told me, oh no, no, no, nothing like that is going to happen even though different rooms had already been allocated per guy and a lady. In fact, I felt everybody understood the plan except me. So, we got into the assigned rooms. I was assigned a room with a total stranger. Thus, for close to an hour, the guy made an advancement for sex which I continued to turn down. I made it clear that I wasn’t going to engage in sex with a person I didn’t know, with that he got violent and started hitting and beating me.

**Theme 3: self-loathing** **as meaning attached to TI experience.**

*I feel useless, knowing I could have fought back. I had been beaten and because that’s basically what happened, I now feel like a complete loser.*

**Theme 5: Divine intervention as the meaning made of tonic immobility.**

*I could not fight off my attacker, but something made me stronger, afterward. I got up and realised that I could walk from there; something somehow gave me my power back. I walked away from the scene and looked for help. God indeed helped me.*

**EXTRACTS FROM TRANSCRIPTS OF SURVIVORS’ STORIES OF THEIR EXPERIENCE FROM TONIC IMMOBILITY**

**PARTICIPANT 6 (31-35).**

My father is a catechist. Usually, my father goes to Father’s house to discuss with him issues concerning the church. And sometimes, I find myself going there because I love the priests and the reverend sisters. And I told my father that I would like to be a reverend sister and my desire moved me to go with my biological father to the Rev. Father’s house. So, this day, I went to the Rev. Father’s house, usually, I go to help, sweep the compound, clean the house, and help in the kitchen, because I liked to understudy the priests and religious. On this day, my biological father did not go with me to visit the Rev. Fr’s house. I went alone and the Rev. Father was home. Hmmmm, it hurts to remember. So, this day, I went there, and the priest welcomed me, but left shortly that he had something to do out. I was busy cleaning his room when he returned shortly after saying he had forgotten his phone but then locked the door behind him and told to me to get his phone from his room. I went to his room to fetch it, only to have Father enter the room and lock the door.

I was wondering why he locked the door as I said Father, this is the phone you asked me to fetch for you. He collected the phone but was coming closer to me, *as he was approaching me, I was retreating backward against the wall, and become deeply scared. I tried to run but, where would I run to? He pounced on me and was hitting me everywhere. He pushed me onto the bed with force, was on top of me, and tore my clothes with such anger and a fierce look in his eyes. I lay frozen with fear on the bed where he pushed me. He stopped hitting me, maybe thinking I was dead, but he continued and raped me repeatedly.* I lost my strength, and I couldn’t scream. I was on the bed, helpless. Father hurt me badly. He raped me until I think, I blacked out. After a while, I regained consciousness, or was I dreaming? I tried to get up from the bed, but I could not, I realized that it was on a hospital bed that I was lying. It hurts to remember.

**Theme 3: self-loathing as meaning attached to TI experience.**

*I see myself now as a second-class citizen, no longer worthy to be a human being. I have nothing to offer; my dignity is gone from me*. Now, I just detest seeing men around me. If they come closer, it’s as if they have a special dirty smell, I just hated men around me, especially the priests. I hate them.

**Theme 4. Suicidal ideation as meaning attached to tonic immobility.**

I am no longer a woman, as my fellow women, as my fellow friends. It was as if that which makes me a woman who has been taken away from me. And so, I see myself as not worth living. At one point, I wanted to commit suicide. I feel, I am not worth living any longer. Especially, *whenever I think about how I just lay down there and failed to protect myself, I feel like I am not worthy of living. I feel like committing suicide. Sometimes I feel like going away forever. Moreover, it has changed everything about me, I am not as free as I used to be, I have become withdrawn, and very sad. So why must I continue to live?*

**EXTRACTS FROM TRANSCRIPTS OF SURVIVORS’ STORIES OF THEIR EXPERIENCE FROM TONIC IMMOBILITY**

**PARTICIPANT 7 (26-30).**

**Theme 2:** **Onset of tonic immobility as self-protection mechanism from further harm**

It happened on a Sunday; May 28 was on a Sunday if I can remember. Around where our shop was, in the evenings often notorious boys from the main market hung around there. Most times to smoke and do drugs. But Grandma’s tailoring shop was at a dark corner of a bad zigzag road. There is a big children’s hospital around the environment. My grandma has had a shop there since before I was born. So, I felt free to go and come from the shop. But that day, so on *my way from my granny’s home, I passed by three men whom I thought were talking among themselves. Immediately I passed them, I felt a hard slap on my back. This was without provocation. The other two joined in beating me. I was overwhelmed and did not struggle with them. They tossed me about before throwing me on the bare ground; and raped me, one after the other. After a while, the beating stopped, I do not know when because I have never been that scared before. I did not try to defend myself; I was shattered. I knew what was happening, but I just did not react because of my fear of them.* I felt embarrassed and sad. I was even disappointed with myself that night. I blamed myself for passing through that road that night. Because even I had heard many stories about people who could be kidnapped in dark lonely streets like that. I see it as if I didn’t learn from that. As they attacked me, I felt weak, irritated I wish I had the power to stop them, but I didn’t. I wasn’t feeling comfortable, the pain. I wished this was not happening. I was still a virgin. My heart was beating so fast as if it was going to stop, and I would just die there and then.

**EXTRACTS FROM TRANSCRIPTS OF SURVIVORS’ STORIES OF THEIR EXPERIENCE FROM TONIC IMMOBILITY**

**PARTICIPANT 8 *(26-30).***

**Theme 2:** **Onset of tonic immobility as self-protection mechanism from further harm**

It [rape] happened with somebody that I liked, whom I was looking forward to being in a relationship with. But we’ve been talking, he has been making mention of us having sex and having very intimate relationship. I have been telling him that I am not ready to get intimate and that if I am ready, I will let him know. So, he was supposed to pick me on a date and go out. On our way, he told me that he needed to get something at his home, that once he got it, we would go on our way to our date. So, I felt ok, after all, it won’t take time and we would soon be on our date. Then we got to his house, I planned to stay in his car, but he was like I should come on in and feel comfortable. So, I said to myself there was no need being uptight since he was not new to me, and he was somebody I was hoping to be in a relationship with. I came down from the car and went into his house. The minute I entered, he quickly locked the door and slipped the key in his pocket. I was surprised he did that. And before I could even process the turn of things all that and ask questions, he had pushed me to the floor and held me down. I was in shock that this was happening. It was just a lot for me to process at that time. In no time, he tore my panties, pulled down his trousers and he raped me. The summary of it is, *he had his way; I was raped. I was not able to shout or do anything, I was just in shock and so confused. I could not believe what was happening at that time to me and by this person. He was a friend. My body felt heavy and motionless on the rug where I had collapsed. At some point, he stopped being violent. It was just a lot for me to process… I remained there, tuned off. I was shaking, sweating, very confused, and crying. I was so lost and confused.* That’s what happened.

**Theme 4. Suicidal ideation as meaning attached to tonic immobility.**

*Not being able to defend myself from the hands of my attacker was the experience that made me have a death wish. It is a terrible experience. After that, I do not see myself as a complete person again; it is like I allowed something to be stolen from me and did not fight for it. I feel like a total mess and cease to exist in my mind after that experience.*

**EXTRACTS FROM TRANSCRIPTS OF SURVIVORS’ STORIES OF THEIR EXPERIENCE FROM TONIC IMMOBILITY**

**PARTICIPANT 9 *(21-25).***

***Theme 5: Divine intervention as the meaning made of tonic immobility.***

Everything kept playing in my head. The thing that was in my head then was just to leave that place or go to the house or just to leave there. So, I found myself on my feet, and I stood up, he was saying, ‘am sorry, am sorry’ and all that. I just had to leave. I felt myself getting very angry, I felt like shouting… like… angry and so I went, left the place, and walked on the road, thinking about everything, like when somebody is drunk, just staggering, walking along the road. I even missed my bus stop, I had to go back. Because everything was just flooding in and that made me confused. I was just thinking about everything. Everything was just coming up in my head. This is not supposed to have happened, it just happened. This made many things go on in my head, and at that moment, I felt like crying, but I couldn’t just cry because I was on the road. So, when I was not even thinking straight, (I- hmmm), I wasn’t even thinking of where I was supposed to go to, that’s why I had to pass my bus stop and I still paid and went back again. But so,

*…after the second experience of a rape that I could not prevent by fighting off the attacker, I could not stand the shame and reminders each time I got home. For this, I decided to relocate to another state from Lagos. It was painful. It was humiliating that I did nothing. But by the grace of God, it helped me decide finally to leave Lagos for another state to continue with my life.* I am ashamed of myself. I just lay there; I did nothing. How can I now express myself in public? That is why I have become withdrawn and keep to myself.

***Theme 3: self-loathing as meaning attached to TI experience.***

*When I remember how I was so helpless and let it happen, the whole thing makes me feel less and less of a human being. It has lowered my self-esteem. I do not even express myself in public anymore. I have been very withdrawn since the incident and now live inside my shadow and my shell.* I have always felt vulnerable. I have always felt like there is this thing that made every guy that sees me want to pounce on me. And I just keep asking and checking myself. Is it that I don’t dress properly, or … I don’t know if there is something about me that always sends wrong signals to men to come and do whatever to me.

**EXTRACTS FROM TRANSCRIPTS OF SURVIVORS’ STORIES OF THEIR EXPERIENCE FROM TONIC IMMOBILITY**

**PARTICIPANT 10 (26-30).**

**Theme 3: self-loathing as meaning attached to TI experience.**

My body felt or reacted to him thus I couldn’t do anything. I was in complete shock, I couldn’t move. I could not fight him back or push him away. I just laid there on the floor, not doing anything so he just had his way. For the experience, at that point, I didn’t really care, all I could think was that this man was taking advantage of me because I was not strong to fight him back or probably it was just us girls in the room and we were helpless. So, he was just doing what he wanted, and I couldn’t fight back so, I was powerless, I didn’t have strength or anything. *I felt bad about myself and the bad things that kept happening to me. And this is not the first time that I’ve been raped. The people around me always had a bad impression of me, which I hate. I hate myself and I do not want to be a burden to anyone.* I remember that when they left, I remained on the floor, I couldn’t stand up or move from where I was. My friend came over and help me up from the floor unto a chair. I was just there. I was just there like I couldn’t do anything. My legs were really shaking. She went to her neighbors and called for help. When they came into the room, I was sitting there like a dummy; I couldn’t do anything. I couldn’t even say a word. I was still in shock.

**EXTRACTS FROM TRANSCRIPTS OF SURVIVORS’ STORIES OF THEIR EXPERIENCE FROM TONIC IMMOBILITY**

**PARTICIPANT 11***(****16-20).***

**Theme 4. Suicidal ideation as meaning attached to tonic immobility.**

**…**I don’t know because they were all beating me, so I do not know. However, I could not resist him. It felt like I was dreaming, I believed that he used a charm on me to make me not fight back. So, I don’t know, but when they are through, I felt pain all over my stomach, I cannot stand up, I was in pains. The situation spelt a lot to me. These days when I see a guy, sometimes I feel the pain all over again, I cannot even talk to a guy normally, even seeing them is rubbish to me.

*When I think and remember that I just lay there and did nothing to defend myself, I feel like killing myself because it is so humiliating. How can I even mention that something like that happened to me, and I did nothing? What am I living for?*

I feel like killing them all. I feel like I have already spoilt my career. During counseling, I asked them whether I still get married. I also asked whether am still a virgin? Can I even tell anyone proudly that I am till a virgin? The story has changed.

**EXTRACTS FROM TRANSCRIPTS OF SURVIVORS’ STORIES OF THEIR EXPERIENCE FROM TONIC IMMOBILITY**

**PARTICIPANT 11** (***26-30).***

I felt bad because, I never hurt anybody, I never do anything bad to anyone…but bad things keep happening to me. I don’t know. When wrong things happen in life, but I don’t know why they happen to me. I feel bad. I lost my virginity through a rape, and it continued because like I couldn’t tell anyone, moreover, the person told me it was my destiny. My first sexual experience was through rape at age 15, I was told it was destined to happen. So, I felt it was destiny in a way. It happened in a Church. My mom’s family are Christians, right? It was in Calabar that I was first raped. I was a child then. This guy who raped me also told me then that, “You will never find somebody better in life”, you are already weird. *That I will never be anything in life because what was happening to me was already written and signed by God and so not a mistake. With the recent rape, I felt deep hate towards myself. I told myself that it was better to die, that I was going to kill myself because I felt I would not be able to heal from all my pains.* That’s why I live my life the way I do, I keep to myself, and I try to make myself happy. I don’t bother about stuff or relationships. Relationships for me do not work, as nothing works. I am always alone, working.

**EXTRACTS FROM TRANSCRIPTS OF SURVIVORS’ STORIES OF THEIR EXPERIENCE FROM TONIC IMMOBILITY**

**PARTICIPANT 13 *(26-30).***

***Theme 5: Divine intervention as the meaning made of tonic immobility.***

It surprised me that he quickly locked the door. I was still wondering why he locked the door while I was there to only collect a cheque when without a word, he pushed me with force unto the bed and was on top of me in no time. He pinned me down, pulled my dress over my head, yanked my underwear off, and entered me. I was totally taken unawares, and it was hard to believe that a man of God would do that. I was totally inactive, I was defenseless, could not even think of what to do, a thousand things running across my mind, I was so scared and was crying also. I was so confused… it was confusing. I tried to struggle but he was heavily on me. I was confused and wondered why me. This is an elderly man of God, and to have done this.

As a Minister of God through songs, I wondered why this was happening to me? I wondered whether God has left me. At some point I just let go, my body just submitted, and my body was tight. I told myself that it was not me, it could not be me (silent tears were streaming down her cheeks now), I did not want to look at him, I think I shut my eyes, I didn’t want to look at him.

My feelings then and now are of sadness. I felt disappointed then as I am now too, it was disbelief to me and still is and I wondered then if it was really happening to me. I still wonder. You know, during the rape, at some point, I just stopped struggling. It was a sad experience*.*

*For days I could not go out. I cancelled all the church activities I had planned for that week, asking myself, “What kind of church activities will I carry out when I don’t feel God’s presence around me anymore? Where was He while I was being raped?” I sometimes think that maybe it was God’s intervention that made me to be still and do nothing in order to protect me****.*** Afterward, I spoke with myself and said, I must push this back this bad thing, and go on with my life.
