# Supplementary material for: I did not scream. i could not; i was terrified. i just followed them. . .i blocked my mind. then they all raped me: A narrative inquiry on the onset of tonic immobility among women rape victims in Nigeria
Source: PLoS One. 2024 Feb 5;19(2):e0278810. doi: 10.1371/journal.pone.0278810 (PMC10843104; doi:10.1371/journal.pone.0278810)
Supplement: Attachment — Submitted filename: Response to Reviewers.docx [file pone.0278810.s003.docx]

**Response to Reviewers**

Subject: PLOS ONE Decision: Revision required [PONE-D-22-32334] - [EMID:7687612a4ba479bd]

**...I did not scream. I could not; I was terrified. I just followed them...I blocked my mind. Then they all raped me: A narrative inquiry on the onset of tonic immobility among women rape victims in Nigeria.**

Please include the following items when submitting your revised manuscript:

A rebuttal letter that responds to each point raised by the academic editor and reviewer(s). You should upload this letter as a separate file labeled 'Response to Reviewers'.

**Author’s Response:**

Done

A marked-up copy of your manuscript that highlights changes made to the original version. You should upload this as a separate file labeled 'Revised Manuscript with Track Changes'.

**Author’s Response:**

Done

An unmarked version of your revised paper without tracked changes. You should upload this as a separate file labeled 'Manuscript'.

**Author’s Response:**

Done

If you would like to make changes to your financial disclosure, please include your updated statement in your cover letter. Guidelines for resubmitting your figure files are available below the reviewer comments at the end of this letter.

**Author’s Response:**

Not applicable

If applicable, we recommend that you deposit your laboratory protocols in protocols.io to enhance the reproducibility of your results. Protocols.io assigns your protocol its own identifier (DOI) so that it can be cited independently in the future. For instructions see: https://journals.plos.org/plosone/s/submission-guidelines#loc-laboratory-protocols. Additionally, PLOS ONE offers an option for publishing peer-reviewed Lab Protocol articles, which describe protocols hosted on protocols.io. Read more information on sharing protocols at https://plos.org/protocols?utm_medium=editorial-email&utm_source=authorletters&utm_campaign=protocols.

**Author’s Response:**

Not applicable

We look forward to receiving your revised manuscript.

Kind regards,

Kornelia Zaręba, MD

Academic Editor

PLOS ONE

**Author’s Response:**

Thank you.

**Journal Requirements:**

When submitting your revision, we need you to address these additional requirements.

1. Please ensure that your manuscript meets PLOS ONE's style requirements, including those for file naming. The PLOS ONE style templates can be found at https://journals.plos.org/plosone/s/file?id=wjVg/PLOSOne_formatting_sample_main_body.pdf and

<https://journals.plos.org/plosone/s/file?id=ba62/PLOSOne_formatting_sample_title_authors_affiliations.pdf>

**Author’s Response:**

We have scrutinized our manuscript to the best of our knowledge to ensure compliance of our manuscript with PLOS ONE’s style requirements.

2. Please include a complete copy of PLOS’ questionnaire on inclusivity in global research in your revised manuscript. Our policy for research in this area aims to improve transparency in the reporting of research performed outside of researchers’ own country or community. The policy applies to researchers who have travelled to a different country to conduct research, research with Indigenous populations or their lands, and research on cultural artefacts. The questionnaire can also be requested at the journal’s discretion for any other submissions, even if these conditions are not met. Please find more information on the policy and a link to download a blank copy of the questionnaire here: https://journals.plos.org/plosone/s/best-practices-in-research-reporting. Please upload a completed version of your questionnaire as Supporting Information when you resubmit your manuscript.

**Author’s Response:** Comments 2 and 3 are the same.

3. Please include a complete copy of PLOS’ questionnaire on inclusivity in global research in your revised manuscript. Our policy for research in this area aims to improve transparency in the reporting of research performed outside of researchers’ own country or community. The policy applies to researchers who have travelled to a different country to conduct research, research with Indigenous populations or their lands, and research on cultural artefacts. The questionnaire can also be requested at the journal’s discretion for any other submissions, even if these conditions are not met. Please find more information on the policy and a link to download a blank copy of the questionnaire here: https://journals.plos.org/plosone/s/best-practices-in-research-reporting. Please upload a completed version of your questionnaire as Supporting Information when you resubmit your manuscript.

**Author’s Response:**

A completed copy of PLOS’ questionnaire on inclusivity in global research is uploaded as Supporting Information I.

4. Please amend your current ethics statement to address the following concerns:

a) Did participants provide their written or verbal informed consent to participate in this study?

b) If consent was verbal, please explain i) why written consent was not obtained, ii) how you documented participant consent, and iii) whether the ethics committees/IRB approved this consent procedure.

**Author’s Response:** Amended. Refer Pages 8 and 10

Yes, ethics committees approved the proposal that prospective participants who were willing to participate in the study would sign consent forms.

5. In your Data Availability statement, you have not specified where the minimal data set underlying the results described in your manuscript can be found. PLOS defines a study's minimal data set as the underlying data used to reach the conclusions drawn in the manuscript and any additional data required to replicate the reported study findings in their entirety. All PLOS journals require that the minimal data set be made fully available. For more information about our data policy, please see http://journals.plos.org/plosone/s/data-availability.

"Upon re-submitting your revised manuscript, please upload your study’s minimal underlying data set as either Supporting Information files or to a stable, public repository and include the relevant URLs, DOIs, or accession numbers within your revised cover letter. For a list of acceptable repositories, please see http://journals.plos.org/plosone/s/data-availability#loc-recommended-repositories. Any potentially identifying patient information must be fully anonymized.

**Author’s Response:** This study’s minimal underlying data set has been uploaded as Supporting Information II

Important: If there are ethical or legal restrictions to sharing your data publicly, please explain these restrictions in detail. Please see our guidelines for more information on what we consider unacceptable restrictions to publicly sharing data: http://journals.plos.org/plosone/s/data-availability#loc-unacceptable-data-access-restrictions. Note that it is not acceptable for the authors to be the sole named individuals responsible for ensuring data access.

We will update your Data Availability statement to reflect the information you provide in your cover letter.

**Author’s Response:** Noted

6. Please review your reference list to ensure that it is complete and correct. If you have cited papers that have been retracted, please include the rationale for doing so in the manuscript text, or remove these references and replace them with relevant current references. Any changes to the reference list should be mentioned in the rebuttal letter that accompanies your revised manuscript. If you need to cite a retracted article, indicate the article’s retracted status in the References list and also include a citation and full reference for the retraction notice.

**Author’s Response:**

i. Reference list number 49

Shrestha, B., & Dunn, L. (2019). The declaration of Helsinki on medical research involving human subjects: a review of seventh revision. *Journal of Nepal Health Research Council*, *17*(4), 548-552

Below is the former reference S/no 49, that has been replaced with the one above for recency.

‘Association WM. WMA Declaration of Helsinki—ethical principles for medical research involving human subjects. 2013’.

ii. This reference ‘Ochberg F. Post-traumatic therapy and victims of violence…’ was left in the reference list as S/no 86 in error. It has been deleted.
iii. Reference no 62 was inserted [PLOS Questionnaire on Inclusivity in Global Research] newly to accommodate the journal requirement S/no 2. With this, reference serial numbers had to change. Former serial no 62, became 63 and this continued until we got the last serial number 113.
iv. Frankl VE. Man’s search for meaning. Rider, S/no 109 was replaced with a more appropriate author - Pattakos, A. (2010). *Prisoners of our thoughts: Viktor Frankl's principles for discovering meaning in life and work*. Berrett-Koehler Publishers.

**Reviewers' comments:**

Comments to the Author

1. Is the manuscript technically sound, and do the data support the conclusions?

The manuscript must describe a technically sound piece of scientific research with data that supports the conclusions. Experiments must have been conducted rigorously, with appropriate controls, replication, and sample sizes. The conclusions must be drawn appropriately based on the data presented.

Reviewer #1: Yes

Reviewer #2: Yes

2. Has the statistical analysis been performed appropriately and rigorously?

Reviewer #1: Yes

Reviewer #2: I Don't Know

3. Have the authors made all data underlying the findings in their manuscript fully available?

The PLOS Data policy requires authors to make all data underlying the findings described in their manuscript fully available without restriction, with rare exceptions (please refer to the Data Availability Statement in the manuscript PDF file). The data should be provided as part of the manuscript or its supporting information or deposited to a public repository. For example, in addition to summary statistics, the data points behind means, medians, and variance measures should be available. If there are restrictions on publicly sharing data—e.g., participant privacy or use of data from a third party—those must be specified.

Reviewer #1: Yes

Reviewer #2: Yes

4. Is the manuscript presented in an intelligible fashion and written in standard English?

PLOS ONE does not copyedit accepted manuscripts, so the language in submitted articles must be clear, correct, and unambiguous. Any typographical or grammatical errors should be corrected at revision, so please note any specific errors here.

Reviewer #1: Yes

Reviewer #2: Yes

5. Review Comments to the Author

Please use the space provided to explain your answers to the questions above. You may also include additional comments for the author, including concerns about dual publication, research ethics, or publication ethics. (Please upload your review as an attachment if it exceeds 20,000 characters)

Reviewer #1: Dear Authors, thank you for the opportunity to review your interesting and feministic, human-oriented qualitative study. The most important value of your research are related to:

1. Depathologization of TI. Thinking in terms of psychiatric conditions might be leading to self-stigma and might be another trigger of self-loathing in many cultures.

**Author’s Response:**

Yes, while agreeing with you the complications relating to self-stigma and self-loathing [as our study also deduced], TI is an emerging phenomenon [Gama et al., 2022], so the term ‘depathologization’ may not particularly apply to it. The phenomenon is regarded as a behavioral state that is characterized by behavioral symptoms rather than a disease (Edelaar et al., 2012; Miyatake et al. 2009).  Thus, it has largely been associated with posttraumatic stress symptoms rather than stemming from a disease entity. [Gama et al., 2022; Magalhaes et al., 2021; Moller et al., 2017]. This is why it was considered the best option not to include participants that in addition were already mentally challenged and those who chose to opt-out were respected [these possibly opted out due to sociocultural factors]. In addition, the findings of the study could serve for the common good, especially in Nigeria where the researchers believed that this study might be the first of its kind. [References are on page 7].

2. Descriptive character of the study might lead to better understanding of TI phenomenon in clinical practice.

**Author’s Response:**

Thank you. It is hoped that the background to the study and the methods section achieves this.

Reviewer #2: I appreciate your work on such a sensitive topic, I have realized that there are few highlighted paragraphs (lines 221-235) and (lines 517-547), is it been revised?

**Author’s Response:** I checked my copy of the submitted manuscript but **could not find the highlighted sections you referred me to**. Kindly, I would appreciate any other guide to help me attend to the comments. Thank you.

I also suggest creating a table for your sample characteristics; present their ages, raped person, or any other data. See page 12.

**Author’s Response:**

Done, table created as Table I. However, participants’ descriptors were limited to Participant number, age range, and type of rape to shroud the identity of the victims.

You have mentioned in your introduction that there are limited knowledge about TI; I think it is important to suggest quantitative studies in the future about the same topic for example "prevalence of TI among rape victims" to find out the about the prevalence and to work forward accordingly.

**Author’s Response:**

Done, refer along line 645, page 28.

6. PLOS authors have the option to publish the peer review history of their article (what does this mean?). If published, this will include your full peer review and any attached files.

**Author’s Response:**

If you choose “no”, your identity will remain anonymous, but your review may still be made public.

**Author’s Response:**

Affirmative

Do you want your identity to be public for this peer review? For information about this choice, including consent withdrawal, please see our Privacy Policy.

Reviewer #1: No

Reviewer #2: Yes: Nazdar Qudrat Abas

[NOTE: If reviewer comments were submitted as an attachment file, they will be attached to this email and accessible via the submission site. Please log into your account, locate the manuscript record, and check for the action link "View Attachments". If this link does not appear, there are no attachment files.]

**Author’s Response:**

Alright

While revising your submission, please upload your figure files to the Preflight Analysis and Conversion Engine (PACE) digital diagnostic tool, https://pacev2.apexcovantage.com/. PACE helps ensure that figures meet PLOS requirements. To use PACE, you must first register as a user. Registration is free. Then, login and navigate to the UPLOAD tab, where you will find detailed instructions on how to use the tool. If you encounter any issues or have any questions when using PACE, please email PLOS at figures@plos.org. Please note that Supporting Information files do not need this step.

**Author’s Response:**

Not applicable

*References to Authors response to Reviewer 1’s comment number 1

Gallup GG, Rager DR. 1996. Tonic immobility as a model of extreme states of behavioral inhibition. In: Sanberg PR, Ossenkopp K-P, Kavaliers M, editors. Motor activity and movement disorders: research issues and applications. New Jersey (NY): Humana Press. p. 57–80

Gama, C. M. F., de Souza Junior, S., Gonçalves, R. M., Santos, E. D. C., Machado, A. V., Portugal, L. C. L., Passos, R. B. F., Erthal, F. S., Vilete, L. M. P., Mendlowicz, M. V., Berger, W., Volchan, E., de Oliveira, L., & Pereira, M. G. (2022). Tonic immobility is associated with posttraumatic stress symptoms in healthcare professionals exposed to COVID-19-related trauma. *Journal of anxiety disorders*, *90*, 102604. https://doi.org/10.1016/j.janxdis.2022.102604

Magalhaes, A. A., Gama, C. M. F., Gonçalves, R. M., Portugal, L. C. L., David, I. A., Serpeloni, F., Wernersbach Pinto, L., Assis, S. G., Avanci, J. Q., Volchan, E., Figueira, I., Vilete, L. M. P., Luz, M. P., Berger, W., Erthal, F. S., Mendlowicz, M. V., Mocaiber, I., Pereira, M. G., & de Oliveira, L. (2021). Tonic Immobility is Associated with PTSD Symptoms in Traumatized Adolescents. *Psychology research and behavior management*, *14*, 1359–1369. <https://doi.org/10.2147/PRBM.S317343>

Miyatake T, Nakayama S, Nishi Y, Nakajima S. 2009. Tonically immobilized selfish prey can survive by sacrificing others. Proc R Soc B Biol Sci. 276:2763–2767.

Möller, A., Söndergaard, H. P., & Helström, L. (2017). Tonic immobility during sexual assault–a common reaction predicting post‐traumatic stress disorder and severe depression. *Acta obstetricia et gynecologica Scandinavica*, *96*(8), 932-938.
